# Aspirin inhibits inflammation and scar formation in the injury tendon healing through regulating JNK/STAT‐3 signalling pathway

**DOI:** 10.1111/cpr.12650

**Published:** 2019-06-21

**Authors:** Yunjiao Wang, Gang He, Hong Tang, Youxing Shi, Xia Kang, Jingtong Lyu, Min Zhu, Mei Zhou, Mingyu Yang, Miduo Mu, Wan Chen, Binghua Zhou, Jiqiang Zhang, Kanglai Tang

**Affiliations:** ^1^ Department of Orthopeadics/Sports Medicine Center, State Key Laboratory of Trauma, Burn and Combined Injury, Southwest Hospital Third Military Medical University Chongqing China; ^2^ Department of Neurology Third Military Medical University Chongqing China

**Keywords:** aspirin, inflammation, tendinopathy, tendon stem cells

## Abstract

**Objectively:**

Tendinopathy is a common problem in sports medicine which can lead to severe morbidity. Aspirin, as the classical representative of non‐steroidal anti‐inflammatory drugs (NSAIDs) for its anti‐inflammatory and analgesic actions, has been commonly used in treating tendinopathy. While its treatment effects on injury tendon healing are lacking, illuminating the underlying mechanism may provide scientific basis for clinical treatment.

**Materials and methods:**

Firstly, we used immunohistochemistry and qRT‐PCR to detect changes in CD14, CD206, iNOS, IL‐6, IL‐10, MMP‐3, TIMP‐3, Col‐1a1, biglycan, Comp, Fibronectin, TGF‐β1，ACAN，EGR‐1 and FMOD. Next, Western blot was used to measure the protein levels (IL‐6, IL‐10, TGF‐β1, COMP, TIMP‐3, STAT‐3/P‐STAT‐3 and JNK/P‐JNK) in TSCs. Then, migration and proliferation of TSCs were measured through wound healing test and BrdU staining. Finally, the mechanical properties of injury tendon were detected.

**Results:**

After aspirin treatment, the inflammation and scar formation in injury tendon were significantly inhibited by aspirin. Still, tendon's ECM was positively balanced. Increasing migration and proliferation ability of TSCs induced by IL‐1β were significantly reversed. JNK/STAT‐3 signalling pathway participated in the process above. In addition, biomechanical properties of injury tendon were significantly improved.

**Conclusions:**

Taken together, the findings suggested that aspirin inhibited inflammation and scar formation via regulation of JNK/STAT‐3 signalling and decreased rerupture risk of injury tendon. Aspirin could be an ideal therapeutic strategy in tendon injury healing.

## 1 INTRODUCTION

Tendons are connective tissues which attach muscle to bone and carry mechanical force, permitting joint and whole body movement.^1^ Repeated over mechanical force loading leads to tendinopathy, which is characterized as imbalance between micro‐tear and repair in tendon. Studies have reported that tendinopathy covers about 30% of sports injuries and its pathogenesis is highly related to inflammation and degradation of connective tissue.^2^, ^3^ Inflammation is a starter and necessary for injury tissue healing process, which may generate scar formation in the injury site. At the same time, scar formation may increase the fragility of tendon, which may induce risk of tendon rupture. Based on this, it is important to treat tendinopathy in anti‐inflammation and inhibiting scar healing process.

NSAIDs inhibit the inflammation and relieve pain through inhibiting prostaglandin. Aspirin, one of the most commonly used NSAIDs for over 150 years, has been recently used to display multiple effects such as antipyretic, analgesic in cardiovascular system,^4^ central nervous system and some cancers.^5^, ^6^ Though aspirin has been used to treat tendinopathy, evidence for this treatment is lacking, especially its effect on tendon regeneration healing.^7^

In response to micro‐damage of tendinopathy, tissues adjacent to the injury area initiate a cascade of inflammation and repair events that are necessary to restore tissue integrity and function. Some reports have pointed out that inflammation reaction has run through the whole process of tissue repair and has played the role like a double sword.^8^ On the one hand, inflammation can induce wound healing and closing through fibrosis and scar formation and prevent wound infection; on the other hand, inflammation‐induced scar healing can hamper tissue regeneration and further may reduce the function of tissue or organ. Inflammation is bound to affect tendon extracellular matrix (ECM). Anomalies in the ECM composition of the scar tissue after tendon injury may contribute to a poor and delayed regeneration healing process resulting in compromised tissue quality^9^; for example, dexamethasone‐induced spontaneous tendon rupture and dexamethasone‐decreased self‐repair capability are very common in clinical practice. TSCs or progenitor cells were reported firstly in human and mouse tendons in 2007 and were confirmed subsequently in rat and rabbit tendons.^10^, ^11^, ^12^ TSCs differed from tenocytes in terms of their colony‐forming ability, self‐renewal ability and multi‐differentiation potential, which enable them to differentiate into tenocytes, adipocytes, chondrocytes and osteocytes,^13^ so the viability and tenogenic differentiation of TSCs are tightly associated with the maintenance of the tendon microenvironment and the development of tendinopathy.

The present study aimed to make clear that the effect of aspirin on TSCs viability in vitro, inflammation and regeneration healing process of tendinopathy and the mechanical properties of the injury tendon, and to provide new therapeutic knowledge of aspirin treatment on tendon injury.

## 2 MATERIALS AND METHODS

### 2.1 Ethics Statement

Eight‐week‐old Sprague Dawley rats weighing 200‐250 g were used and housed under a 12 hours light/dark cycle in a pathogen‐free area with free access to water and food. All animals were treated according to institutional guidelines for laboratory animal treatment and care. All experimental procedures were approved by the Animal Research Ethics Committee of the Third Military Medical University, China.

### 2.2 Animal model establishment

A total of 24 male Sprague Dawley rats (8 weeks old, 200‐250 g) were divided into three groups: the intact group (C), the injury group and aspirin treatment group after injury model establishment (ASA group). In injury and ASA groups, rats were injected with 30 μL type I collagenase solution (10 mg/mL) into both Achilles tendons to establish micro‐damaged Achilles tendon model. In ASA group, aspirin (30 mg/d) was given to each rat through gavage administration. One week after establishment of injury model, aspirin was given to each rat in ASA group.

### 2.3 Isolation and culture of rat TSCs

Isolation of rat TSCs was performed as our previously described.^14^ Briefly, the Achilles tendons from both hind feet were dissected after euthanasia. Only the mid‐substance tissue was collected, and peritendinous connective tissue was carefully removed. Mid‐substance tissue was minced in sterile phosphate‐buffered saline (PBS) and digested in 3 mg/mL of type I collagenase (Sigma‐Aldrich, St. Louis, MO, USA), 2.5 hours at 37℃. A 70 mm cell strainer (Becton Dickinson, Franklin Lakes, NJ, USA) was used to yield a single‐cell suspension. The released cells were washed in PBS, centrifuged at 300 g for 5 minutes and then resuspended in Dulbecco's modified Eagle's medium (DMEM) (Gibco, Carlsbad, CA, USA) with 10% foetal bovine serum, 100 U/mL penicillin, 100 mg/mL streptomycin and 2 mmol/L L‐glutamine (all from Invitrogen, Carlsbad, CA, USA). Isolated cells were plated and grown for 2 days at 37℃ in 5% CO2 and then washed twice in PBS to remove non‐adherent cells. On day 7 of culture, the cells were trypsinized with trypsin‐EDTA solution (Sigma‐Aldrich), mixed together and cultured as passage 0 cells. Cells from passages 3 (P3) were used in the subsequent experiments. TSCs were seeded onto 6‐well plates for real‐time quantitative PCR (qRT‐PCR) and scratch assays, and 10 cm diameter Petri dishes for protein extraction.

TSCs were seeded into a six‐well plate at a density of 6 × 10^4^ cells/well for each experiment. We added IL‐1β (10 ng/mL) as pro‐inflammatory stimulation to establish inflammatory cell model in vitro. To evaluate effect of aspirin on TSCs, we applied IL‐1β alone, aspirin (2 mmol/L) alone and IL‐1β + aspirin (2 mmol/L) for 24 hours in addition to no‐treatment control group. In inhibition studies, TSCs were pre‐incubated with JNK inhibitor SP600125 (10 μmol/L) or STAT‐3 inhibitor S3I‐201 (100 μmol/L) for 1 hour before addition of IL‐1β + ASA for 24 hours. Culture medium with or without agents was changed every 3 days throughout the experiments.

### 2.4 Histomorphometry

The Achilles tendon specimens were fixed in 4% paraformaldehyde. Nine sections were cut at a thickness of 4 mm and stained with haematoxylin and eosin. To analyse the changes of total histological scores on HE‐stained slides after treatment, we used the established histological scoring system by Stoll 15 et al The score of the intact group was defined as 20 points.

### 2.5 Immunostaining

At week 4 after aspirin treatment, eight rats each group were sacrificed. The Achilles tendons were harvested for immunohistochemistry. Serial sagittal paraffin sections were prepared from the Achilles tendons as previously described.^16^ Briefly, the sections were heated at 60℃ for 1 day. After deparaffinization, sections were blocked through H_2_O_2_ and methanol for 15 minutes under dark light. After that, sections were subjected to antigen retrieval through pepcase and tryptase for 30 minutes in 37℃. After washed in PBS for 5 minutes, sections were incubated in goat serum for 30 minutes. After discarding the serum, sections were incubated with inflammatory and tissue repair‐related markers iNOS (Proteintech, 1:200), CD14(Proteintech, 1:200), CD206(Proteintech, 1:200), IL‐6(Proteintech，1:200), IL‐10(Proteintech,1:200), MMP‐3(Proteintech,1:200) and TIMP‐3(Proteintech,1:200) at 4℃ overnight. After rewarming the sections at room temperature for 30 minutes, sections then were washed by PBS for 4 times every 5 minutes. Sections were incubated with secondary antibodies (Bioss, 1:200) for 20 minutes and treated with horseradish peroxidase for 20 minutes. Sections were added with DAB for about 1 minute under microscope observation. Washed slightly in water for 10 minutes, sections were treated with haematoxylin for about 30 seconds. Then sections were washed slightly in water for 10 minutes and heated in 60℃ water for 15 minutes. At last, sections were dehydrated and mouted.

Serial coronal frozen sections (5 mm) were prepared from the Achilles tendons as previously described.^1^ Briefly, sections were rewarmed at room temperature for 30 minutes and washed in PBS for 4 times every 5 minutes. With punching and blocking the sections with 0.1% Triton X‐100 and 5% BSA for 1 hour, sections were incubated with scar formation‐related markers COMP (Proteintech, 1:200), FMOD (Bioss,1:200), biglycan (Proteintech,1:200) and Fibronectin (Proteintech, 1:200) at 4℃ overnight. Sections were incubated with goat anti‐rabbit IgG H&L (Dylight‐594) (Proteintech, 1:200) for 1 hour. After washed for 4 times every 5 minutes, sections were counterstained with (4',6‐diamidino‐2‐phenylindole, DAPI) for 10 minutes. At last, sections were mounted by fluorescence quencher.

### 2.6 Real‐time quantitative PCR

The mRNA expression levels of tendon‐related genes were determined using qPCR. Total RNA was extracted from cells using TRIzol reagent, according to the protocol provided by the manufacturer (Takara, Dalian, China). cDNA was synthesized from total RNA using a Superscript III First‐Strand Synthesis Kit (TaKaRa). qPCR was performed using a SYBR Green RT‐PCR kit (TaKaRa) and an ABI Prism 7900 Sequence Detection System (PE Applied Biosystems, Foster City, CA, USA). Expression levels were calculated relative to the expression of the housekeeping gene glyceraldehyde 3‐phosphate dehydrogenase (GAPDH).

### 2.7 Migration analysis

Migration analysis was performed similarly to the previous studies.^17^, ^18^ For random migration, 6.0 × 10^4^ cells of TSCs were seeded on 6‐well plates. Marker lines were made behind the plates, and at least five lines were on the every well. On the next day, the tips were used to scratch the lines which are perpendicular to the marker lines on the wells; then, serum‐free medium was added into the wells. Time‐lapse photography was performed at 0, 3, 6, 16, 24 and 36 hours. Scratch coverage area was analysed with ImageJ at every time point. Results of random TSCs migration were measured by three independent analyses.

### 2.8 Protein extraction and Western blotting

The cells were washed twice with PBS and lysed in lysis buffer (50 mmol/L Tris‐HCl, pH 8.0, 1 mmol/L EDTA, 1% Triton X‐100, 0.5%sodium deoxycholate, 0.1% sodium dodecyl sulphate (SDS), 150 mmol/L NaCl) containing a mixture of proteinase inhibitors (Thermo Fisher Scientific Inc, Rockford, IL, USA). Total protein concentrations were measured using a BCA Protein Assay Kit (Thermo Fisher Scientific Inc, Rockford, IL, USA), and equal amounts of proteins samples (30 µg/lane) were resolved by SDS‐polyacrylamide gel electrophoresis and then transferred onto polyvinylidene difluoride membranes, and membranes were blocked by incubating with 5% non‐fat milk containing 0.1% TBS‐Tween for 2 hours at room temperature. The membranes were then incubated sequentially with primary antibodies overnight at 4℃. The following primary antibodies were used: anti‐STAT‐3 (Proteintech,1:2000), anti‐phospho‐STAT‐3 (cell signalling technology, 1:2000), anti‐JNK (Proteintech,1:2000), anti‐phospho‐JNK (Bioss,1:2000), anti‐TIMP‐3 (Proteintech,1:2000), anti‐IL‐6 (Bioss,1:2000), anti‐IL‐10 (Bioss,1:2000), anti‐TGF‐b (Proteintech,1:2000) and anti‐COMP (Proteintech,1:2000); GAPDH (Proteintech,1:5000) was used as an internal control. Following primary antibody incubation, membranes were washed 3 times in 0.1% TBST and incubated in goat anti‐rabbit IgG (H&L)‐HRP conjugate (Proteintech, 1:2000) 2 hours at room temperature. Proteins were visualized and images captured using a LiCor Odyssey Imager (LI‐COR Biosciences, Lincoln, NE, USA).

### 2.9 Biomechanical analysis

We followed the procedures as described in the previous study.^19^ The Achilles tendon with bony end was first isolated. The two bony ends of tendon were fixed on a custom‐made testing jig with two clamps. The lower one was used to fix the calcaneus end, while the upper one was used to fix the tibia end. The whole construct was then mounted onto the biomechanical testing machine.

### 2.10 Statistical analysis

All values were expressed as mean ± standard deviation (SD). Student's *t* test was used to compare between two groups. Multiple comparisons were made using a one‐way analysis of variance followed by Fisher's tests. A *P*‐value of <0.05 was considered to be statistically significant.

## 3 RESULTS

### 3.1 Aspirin improves tendon healing and changes abnormal macrophage profile in tendinopathy

**Figure 1:**
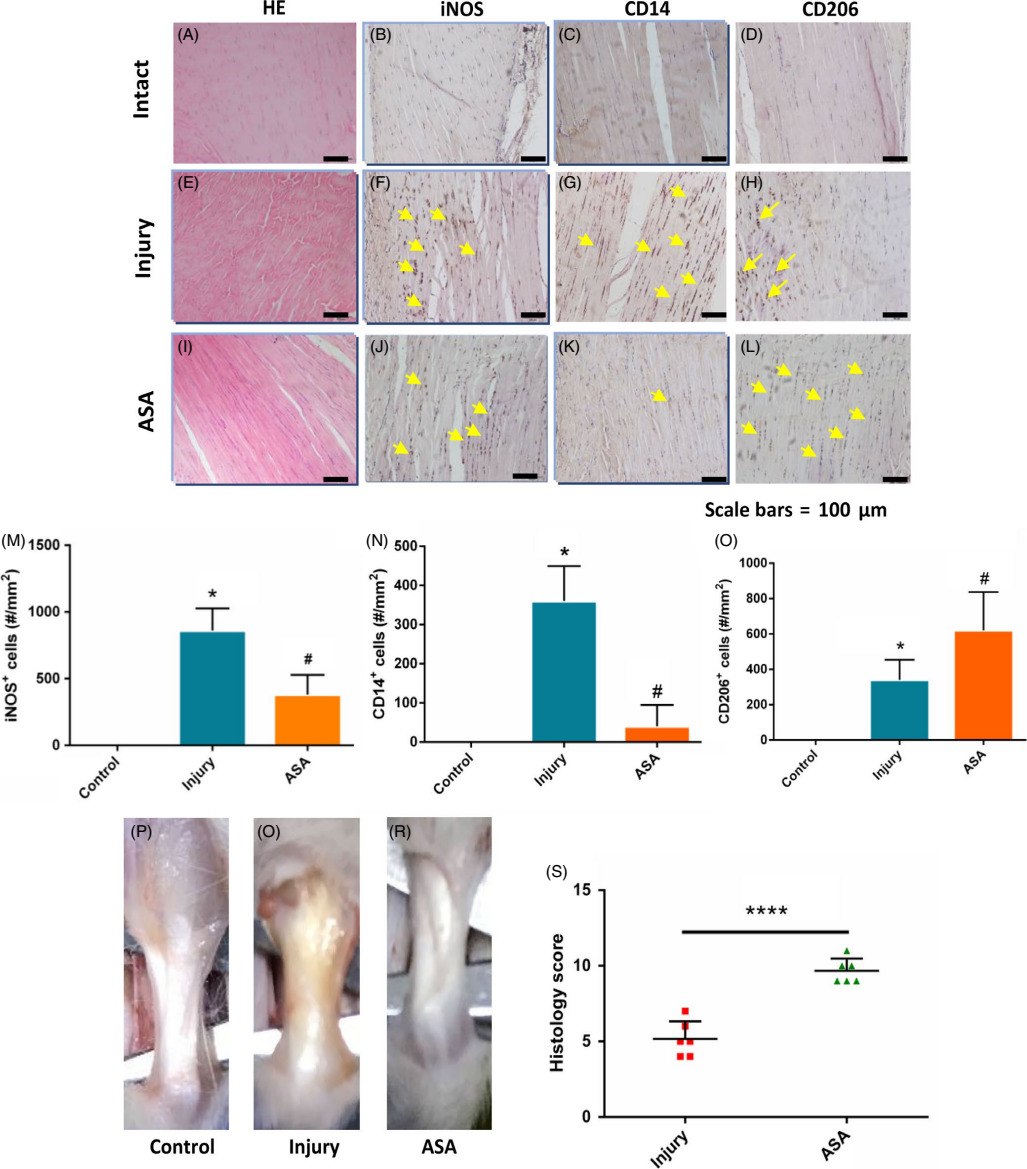
Aspirin changes abnormal macrophage profile in rat tendinopathy model and improve tendon healing. More continuous and integrated tendons occurred in ASA treated group (I) compared with injury group (E). iNOS + M1 Mφs and CD14+ monocytes were more abundant in injury (F,G) compared with ASA treated tendon (J,K). CD206+ M2 Mφs showed reverse tendency compared with iNOS + M1 Mφs and CD14 + monocytes(H,L). The tendons in ASA treatment group were thinner compared with injury model group(P‐R). Histology score showed that score of ASA treatment group was higher than those of injury group(S). The data are presented as the means ± SD. *: vs control, #: vs injury group, N = 6

Haematoxylin and eosin staining revealed that tendon in ASA treatment group showed more continuous and integrated phenotype compared with discontinuous tendon in injury group (Figure 1A,E,I). To verify the effect of aspirin on tendinopathy, we observed the number of changes in iNOS^+^M1 Mφs, CD14^+^ monocytes and CD206^+^ M2 Mφs. There was little expression of iNOS, CD14 and CD206 in the intact control group (Figure 1B‐D). One week after collagenase injection, there was a intense expression of iNOS−, CD14− and CD206‐positive in the injury tendon compared with intact tendon (Figure 1F‐H). The expression of iNOS and CD14 decreased and CD206 increased (Figure 1J‐L) at week 4 after ASA treatment. The rat tendinopathy animal model was established successfully. Tendons in injury group were thicker than those in control group and ASA treatment reversed the thick tendons in the injury group (Figure 1P‐R). Histological score of ASA treatment increased significantly compared with injury group (Figure 1S).

### 3.2 Aspirin inhibits IL‐6 and promotes IL‐10 expression of injury tendon

**Figure 2:**
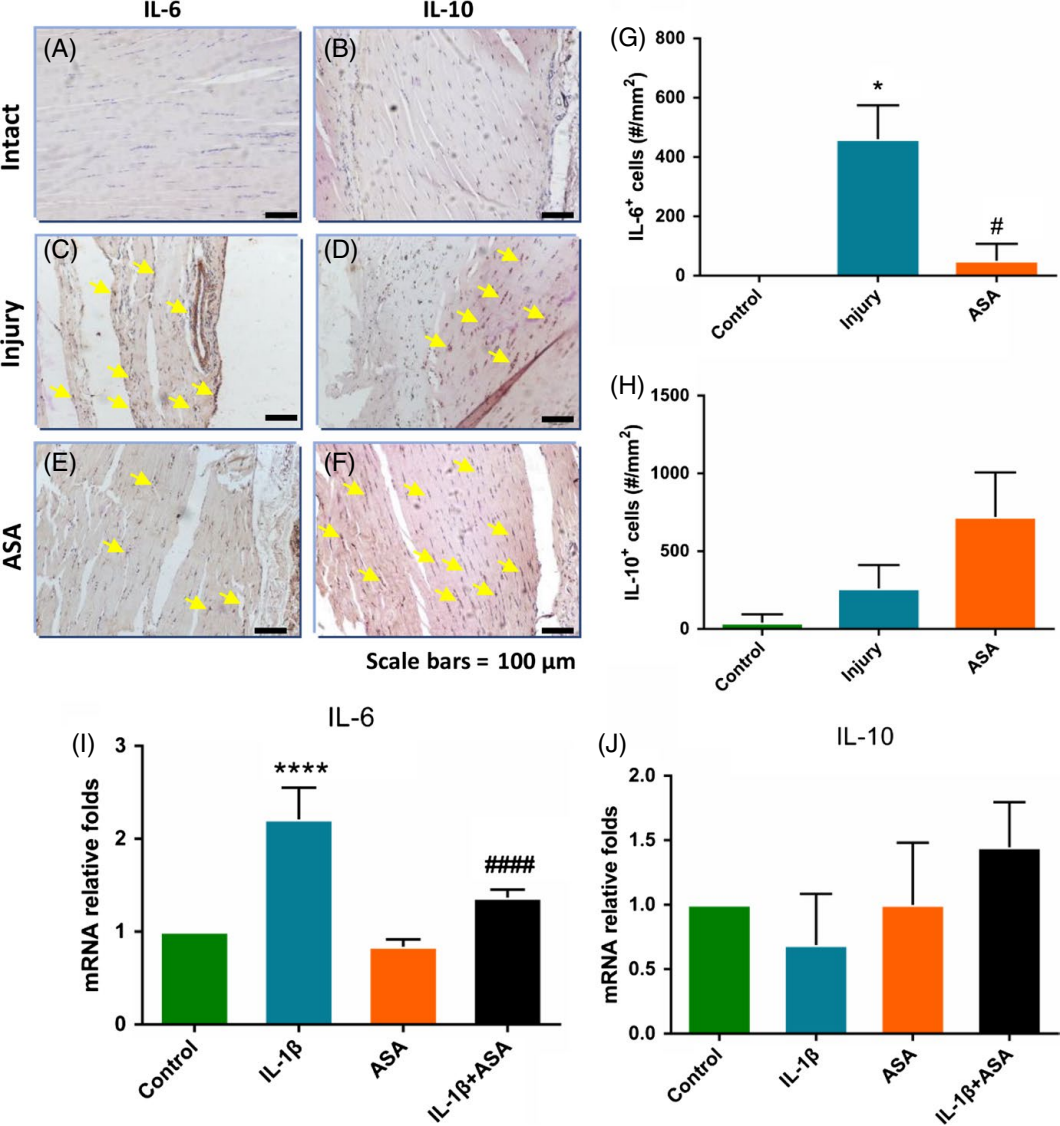
Aspirin changes IL‐6 and IL‐10 expression in tendon healing at 8 weeks after tendinopathy model establishment (A‐F). Quantitatively, there were significantly fewer IL‐6 + cells (G) and more IL‐10 + cells (H) with ASA treatment group compared with injury group (C, D). qRT‐PCR also showed that aspirin treatment lowered expression of IL‐6(I) and elevated expression of IL‐10(J) compared with those in IL‐1β group. The data are presented as the means ± SD. *: vs control, #:vs injury or IL‐1β group, N = 6

Next, we explored the effect of aspirin on pro‐inflammatory factor IL‐6 and anti‐inflammatory factor IL‐10. The results of immunohistochemistry showed that the expression of IL‐6 and IL‐10 in injury group increased significantly compared with intact tendon control group (Figure 2A‐D). After ASA treatment, the expression level of IL‐6 decreased and that of IL‐10 increased (Figure 2E‐F). Similarly, gene expression in vitro showed that ASA treatment significantly decreased the expression of IL‐6 induced by IL‐1β, and IL‐10 expression was elevated by ASA treatment (Figure 2I‐J).

### 3.3 In vivo* aspirin improves tendon healing through changing expression of MMP‐3, TIMP‐3 and Col‐1a1*

**Figure 3:**
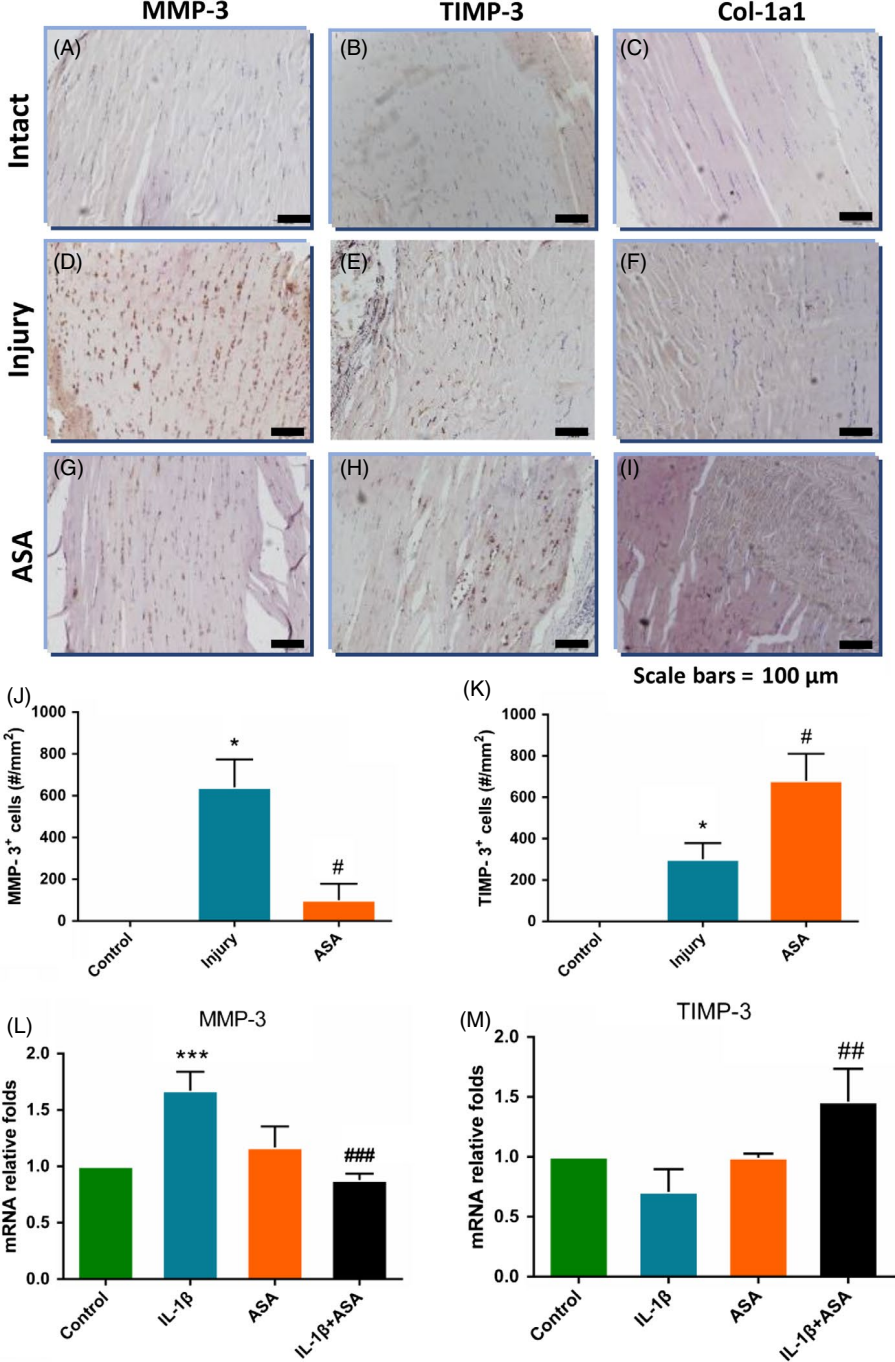
MMP‐3, TIMP‐3 and Col‐1a1 expression in tendon healing mediated by aspirin at 4 weeks after tendinopathy model establishment. Without aspirin, MMP‐3 was predominantly expressed in healing junction (D), whereas aspirin‐delivered tendons showed attenuated MMP‐3 expression along with reorienting collagen fibrils (G). Similarly, level of TIMP‐3 expression was increased in aspirin‐delivered tendon (H) compared with control (E). There was no statistical difference of Col‐1a1 between injury group (F) and aspirin treated group (I). qPCR showed elevated TIMP‐3 and Col‐1a1 expression upon ASA treatment and decreased expression of MMP‐3 in ASA treatment (L‐N). The data are presented as the means ± SD. *: vs control, #: vs injury or IL‐1β group, N = 6

Then, we discussed the effect of aspirin on tendon matrix‐related factors, matrix metalloproteinase‐3(MMP‐3), tissue inhibitors of metalloproteinase‐3(TIMP‐3) and Col‐1a1. The expression of MMP‐3, TIMP‐3 and Col‐1a1 in injury group significantly increased compared with the control group (Figure 3A‐F), while, after ASA treatment, expression of MMP‐3 which accelerates extracellular matrix dissolution decreased (Figure 3G). The expression of tissue‐promoted factors TIMP‐3 and Col‐1a1 in ASA treatment group became larger than injury group (Figure 3H‐I). qPCR analysis demonstrated the similar trends as those in vivo (Figure 3L‐N).

### 3.4 In vitro* aspirin reduces scar formation‐related gene expression and reduces formation of scar tissue *in vivo

**Figure 4:**
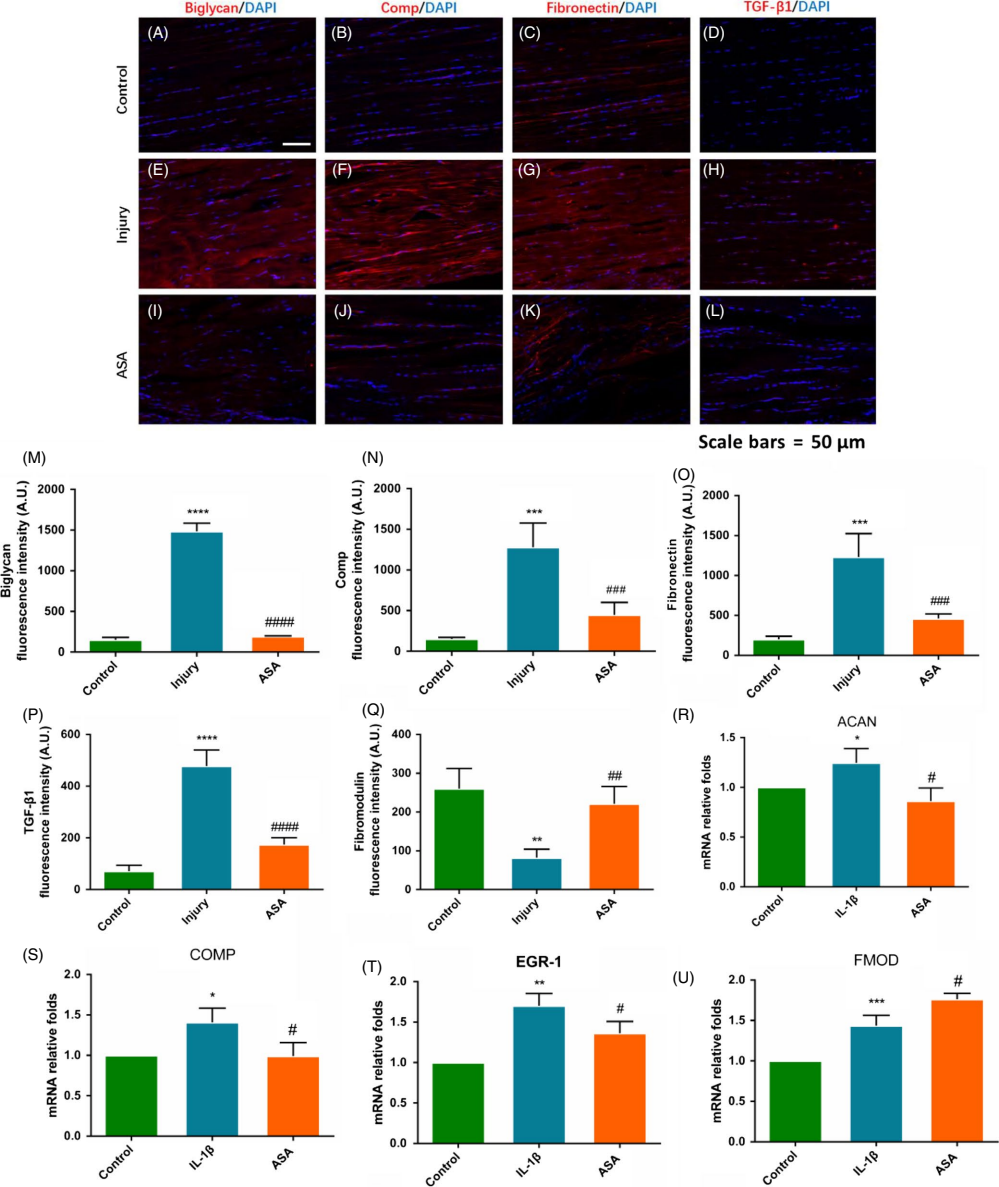
Aspirin altered expression of scar formation in vivo and vitro. (A‐Q) ASA treated tendons displayed significantly lower expression levels of scar formation markers for biglycan, COMP, Fibronectin and TGF‐β1. (R‐U) Consistently, scar formation gene markers for ACAN, COMP and EGR‐1 were reduced by ASA treatment, and anti‐scar formation marker for FMOD was elevated. The data are presented as the means ± SD. *: vs control, #: vs injury or IL‐1β group, N = 6

After that, we discussed the changes in scar formation after ASA treatment. In vivo, expression levels of scar formation‐related gene markers of biglycan, Fibronectin, Comp and TGF‐β1 in ASA treatment group were significantly lower than those in Col‐1a1 group (Figure 4A‐Q). The expression levels of scar formation‐related gene markers in ASA+ Col‐1a1 group were further evaluated by quantitative RT‐PCR in vitro. The results showed that scar formation‐related markers ACAN, COMP and EGR‐1 decreased after ASA treatment, and anti‐scar formation marker FMOD increased (Figure 4R‐U).

### 3.5 In vitro* aspirin reduces cell migration and proliferation of TSCs stimulated by IL‐1β*

**Figure 5:**
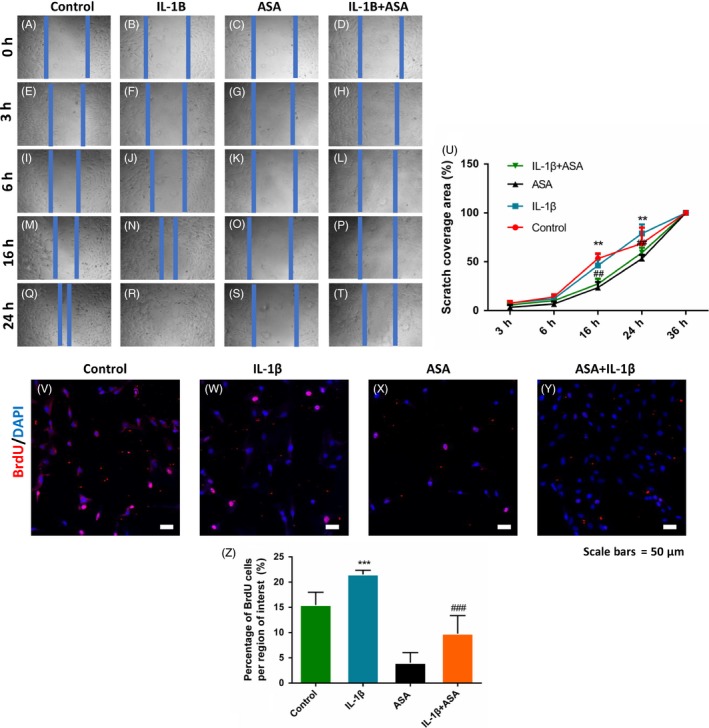
Aspirin significantly reduced TSCs migration and proliferation. (A‐U) In vitro wound healing assays showed that scratch closure of ASA + IL‐1β group was significantly slower compared with IL‐1β treated TSCs. (V‐Z) BrdU assay showed that ASA + IL‐1β group diminished TSCs proliferation induced by IL‐1β. The data are presented as the means ± SD. *: vs. control, #: vs. injury group, N = 3

Migration and proliferation were tested through scratch assay and BrdU staining. Firstly, we found that TSCs motility increased significantly after IL‐1β stimulation. While, to our surprise, ASA+ IL‐1β delayed the migration of TSCs compared with that in IL‐1β group (Figure 5A‐U). Secondly, IL‐1β stimulated TSCs proliferation and ASA + IL‐1β reversed this promotion effect on TSCs (Figure 5V‐Z).

### 3.6 JNK/STAT‐3 signalling is engaged in aspirin‐induced anti‐inflammation and tendon healing

**Figure 6:**
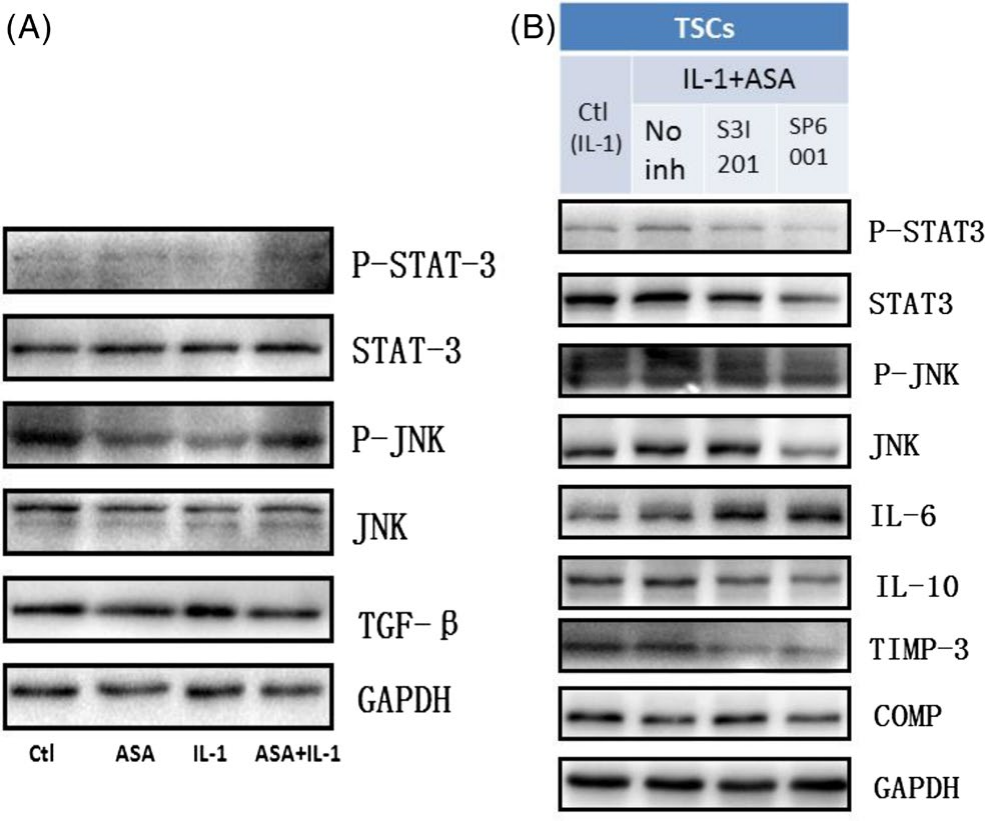
JNK/STAT‐3 signalling is engaged in the aspirin‐induced anti‐inflammation and tendon healing. JNK and STAT‐3 signalling involved in aspirin‐induced IL‐10 and TIMP‐3 expression. Western blot analysis revealed P‐STAT3 and P‐JNK in TSCs upon ASA and IL‐1β for 24 h (A). ASA + IL‐1β induced STAT‐3 and JNK phosphorylation and inhibited TGF‐β signalling. The results revealed that effects of STAT‐3/JNK inhibitors on inflammation and ECM balancing (B). S3I‐201 inhibited STAT3 phosphorylation, whereas SP600125 inhibited phosphorylation of JNK. In contrast, consistently, IL‐10 and TIMP‐3 expressions induced by ASA + IL‐1β were significantly diminished by STAT3 and JNK signalling inhibitors, while decreasing expression of COMP was significantly reversed by S3I201 only. N = 3

Western blotting showed that ASA + IL‐1β group increased P‐STAT‐3 and P‐JNK compared with IL‐1βonly group, and expression of STAT‐3 and JNK had no differences between IL‐1β group and IL‐1β + ASA group (Figure 6A). After adding the STAT‐3 inhibitor S3I‐201 and JNK inhibitor SP600125, the increase trend of P‐STAT‐3 and P‐JNK was reversed by two inhibitors. At the same time, IL‐10 and TIMP‐3 expressions in TSCs induced by IL‐1β + ASA were significantly diminished by STAT3 and JNK signalling inhibitors, and IL‐6 was significantly promoted by the two inhibitors, while decreasing expression of scar formation marker COMP was significantly reversed by S3I201 only (Figure 6B).

### 3.7 Aspirin improves tendon healing and biomechanical character in injury tendon

**Figure 7:**
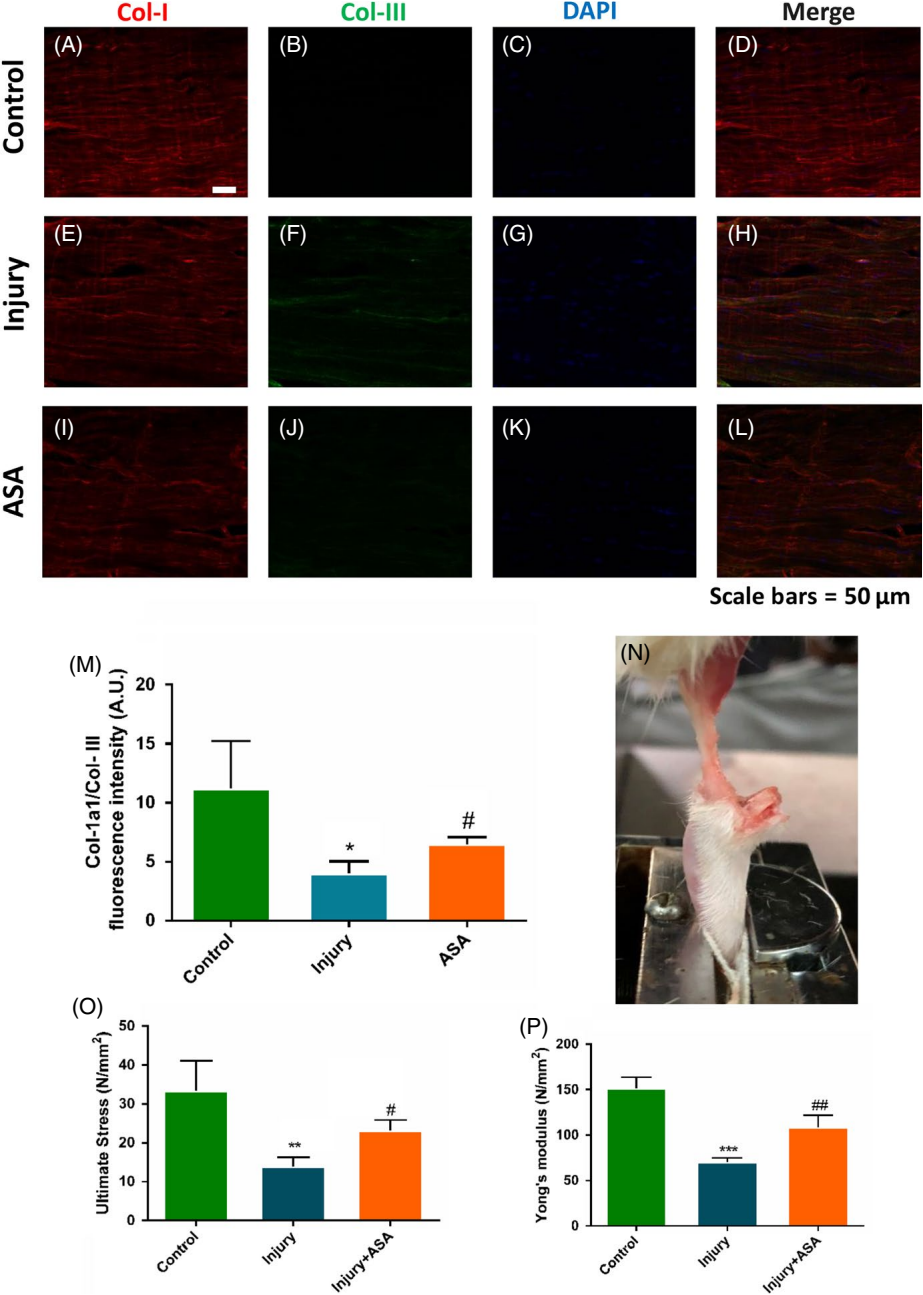
Aspirin treatment on tendon healing in rat Achilles tendon injury model. At 4 weeks after ASA treatment, the tendon samples were collected for biomechanical testing and healing analysis. (A‐M) Col‐1a1/Col‐III increased significantly in ASA treatment group comparing with injury group. (N‐P) The biomechanical properties were improved significantly by ASA treatment after injury. The data are presented as the means ± SD. *: vs control, #: vs injury group, N = 6

Four weeks after ASA treatment on tendinopathy, the tendon samples were collected for tendon healing analysis and biomechanical testing. Col‐1a1/Col‐III which was representative of tendon healthy conditions increased significantly in ASA treatment group comparing with injury group (Figure 7A‐M). The biomechanical testing results showed that the ultimate stress and Young's modulus were significantly higher in ASA treatment group compared with those in injury group (Figure 7N‐P).

## 4 DISCUSSION

To speed up healing process, especially regeneration healing process of tendon injuries is still a big challenge because of the poor understanding of tendon compared with the other components of the musculoskeletal system, and especially the molecular mechanisms controlling the migration, proliferation and fate of TSCs during tendon repair are not well understood.^20^, ^21^ NSAIDs inhibit the function of the cyclooxygenase enzymes (COX‐1 and/or COX‐2), which catalyse the formation of prostaglandins. The inhibition of prostaglandin dampens inflammation and pain, and NSAIDs are effective at relieving symptoms in conditions such as rheumatoid arthritis. Though ASA and especially its ramification have been widely used for the treatment of tendinopathy in clinical practice, few reports have shown the effect of aspirin on scar formation and injury tendon healing process.

The present study demonstrated for the first time that aspirin modulated inflammation and ECM's balance in the injury tendon healing process. On the one hand, aspirin mobilized and stimulated anti‐inflammatory M2 and inhibited pro‐inflammatory M1. On the other hand, aspirin improved expression of anti‐inflammatory IL‐10 and tissue repair‐related TIMP‐3, and decreased expression of pro‐inflammatory IL‐6 and ECM degradation‐related MMP‐3. The results suggested the anti‐inflammation and pro‐regeneration effect of aspirin on tendinopathy. There are two main macrophage subsets M1 and M2, named according to (helper T cells，Th) Th1‐ or Th2‐type responses. M2 is a kind of anti‐inflammatory macrophage cells, which is associated with many diseases such as immune responses of parasites, wound healing and tissue remodelling. Pro‐inflammatory M1 macrophage cells are not only associated with infectious diseases and inflammatory diseases, but also related to metabolic diseases such as atherosclerosis and insulin resistance. Monocytes are precursor cells of macrophages, which can be polarized to M1 or M2 macrophage cells. Reports have shown that macrophage depletion reduces cell proliferation and extracellular matrix (ECM) accumulation and ultimately increases the tensile strength of the injured Achilles tendon 22D. The results showed that aspirin may inhibit inflammation and promote tissue remodelling through increasing M2 and decreasing M1 cells.

IL‐6 is a kind of interleukin which plays both pro‐inflammatory and anti‐inflammatory effects. Interleukin 6 is secreted by T cells and macrophages to stimulate immune response during infection or trauma, especially burns or other tissue damage leading to inflammation. IL‐6 also plays a role in fighting infection in bacterium Streptococcus pneumonia mice.^23^ Interleukin 10 (IL‐10), also known as human cytokine synthesis inhibitory factor (CSIF), is an anti‐inflammatory cytokine. In addition to IL‐10 itself, this group of cytokines encompasses IL‐19, IL‐20, IL‐22, IL‐24 and IL‐26, which are collectively referred to as the IL‐20 subfamily, as well as the more distantly related members IL‐28A, IL‐28B and IL‐29, also known as the interferon (IFN)‐λ family or type III IFNs.^24^, ^25^, ^26^ It downregulates the expression of Th1 cytokines, MHC class II antigens and co‐stimulatory molecules on macrophages. It also enhances B‐cell survival, proliferation and antibody production. IL‐10 can block NF‐κB activity and is involved in the regulation of the JAK‐STAT signalling pathway through binding IL‐10 receptors 1 and 2.^27^ In cytokines aspects, aspirin may execute its anti‐inflammation through inducing IL‐10 and decreasing IL‐6.

MMP‐3 is a member of MMP family, which is involved in the breakdown of extracellular matrix proteins and tissue remodelling in normal physiological processes or disease processes, such as arthritis, and tumour metastasis.^28^, ^29^ TIMP‐3 is member of tissue inhibitor of metalloproteinases family, which is a group of peptidases involved in degradation of the ECM. TIMP‐3 and MMP‐3 are a pair of mutual antagonism to balance the ECM's metabolism.^30^ The present study showed that ASA treatment balanced tendon ECM synthesis and degradation through modulating the metabolic balance between MMP‐3 and TIMP‐3.

The ECM of tendon tissue consists of collagen I, collagen III, elastin and various proteoglycans and mucopolysaccharides.^16^ Erroneous ECM deposition in scar tissue after tendon injury may lead to a poor and delayed healing process resulting in compromised tissue quality.^9^ Promoted by the observation, we observed that ECM‐related proteins were changed in ASA treated group. Biglycan is a kind of proteoglycan which is found in a variety of extracellular matrix tissues. COMP**,** an ECM protein primarily present in cartilage, is present in high quantities in fibrotic scars and systemic sclerosis.^31^ FMOD participates in the assembly of the collagen fibres of the extracellular matrix. It regulates TGF‐β activities by sequestering TGF‐β into the extracellular matrix, which inhibits the scar formation.^32^ Fibronectin has profound effects on wound healing, and it is located in the extracellular matrix of embryonic and adult tissues. It plays a major role to assist fibroblast to adhere to fibrin. The increased ECM scar‐related protein deposition reminded us of inhibition effect on scar formation of ASA treatment.

Surprisingly, we accidentally found that ASA's adding may hinder TSCs migration and proliferation induced by IL‐1β. We supposed that inhibition of inflammation reduced inflammatory factors and that may be related to decreasing TSCs assembling to injury site. Decreased number of TSCs may reduce the ECM deposition in injury tendon. In return, decreased ECM secretion may reduce the scar formation. These can explain that aspirin improved the tendon healing but inhibited the migration and proliferation of TSCs. More studies should be needed for balancing ASA's anti‐inflammation and anti‐scar formation effects and reducing proliferation of TSCs.

STAT‐3 is downstream of a number of cytokines, including IL‐6, IL‐17, IL‐21 and IL‐23.^33^ At the same time, STAT‐3 is necessary for exhibition of anti‐inflammatory effects of IL‐10 34, 35, 36 and IL‐10 production.^37^, ^38^ Similarly, STAT3 signalling has been identified in cancer inflammation, neuronal differentiation 39 and in tendon healing or pathology.^40^ Our signalling data presented a novel role of STAT3 signalling axis in ASA‐induced IL‐10 and TIMP‐3 expression of TSCs, and that were related to ASA‐inhibited tendinopathy inflammation and scar formation.

Inflammation is a pivotal process in both normal and pathologic tendon healing. There is substantial evidence in different model organisms that the immune system is of primary importance in determining the quality of the repair response, including the extent of scarring, and the restoration of organ structure and function.^41^ Like a double‐edged sword, inflammation warranted the normal scar healing, but excessive inflammation may lead to excessive scar formation. Just like excessive scar formation after Caesarean section in uterus induced hysterorrhexis, excessive inflammation may lead to the Achilles tendon rupture. So the present study aimed to explore whether inflammation inhibition can decrease scar formation and the risk of the Achilles tendon rupture. To evaluate the effect of decreased scar on tendinopathy healing, we evaluated the expression of Col‐III and Col‐I. The ratio of Col‐III and Col‐I represented the health condition of tendon. We lastly examined the biomechanical properties in injury tendon after ASA treatment. The results showed that the breaking strength and Yong's modulus increased in ASA treatment group compared with those of injury group. The results reminded us that aspirin decreased rupture risk of the Achilles tendon.

In summary, the present study demonstrated that aspirin inhibited the inflammation and scar formation in tendinopathy tendon, and the process was related to JNK/STAT‐3 signalling pathway. On the other hand, aspirin decreased migration and proliferation ability, which explained that aspirin decreased the excess ECM's deposition. In our previous study, we demonstrated that high concentration of aspirin can induce TSCs apoptosis via inhibiting Wnt/β‐Catenin pathway.^42^ The present study demonstrated that the proper concentration of aspirin regulated inflammation and scar formation in the injury tendon healing process, providing new therapeutic evidence of aspirin on tendon injury.

## AUTHORS' CONTRIBUTIONS

YJW participated in the animal experiment, histological experiment, experimental design, acquisition of data, data analysis and interpretation, and manuscript writing. GH, YXS and HT acquired the experimental data of the immunostaining, Western blot and qRT‐PCR. MZ, XK and JTL joined the experimental design and manuscript revision. MZ, MYY and MDM contributed to the experimental design. WC and BHZ modified grammar and polished the manuscript. JQZ took part in the conception and design. KLT conducted the conception, design and manuscript writing. All authors read and approved the final manuscript.

## COMPETING INTERESTS

No competing financial interests exist among any authors in relation to this submission.

## ETHICS APPROVAL AND CONSENT TO PARTICIPATE

All experimental procedures were performed in accordance with the National Institutes of Health (NIH) Guidelines for Care and Use of Laboratory Animals and approved by the animal research ethics committee of Third Military Medical University.

## CONSENT FOR PUBLICATION

Not applicable.

## Data Availability

The authors declare that the data supporting the findings of this study are available within the article and its supplementary information files.
